# Synthesis and Properties of Ba_6_Fe_2_Te_3_S_7_, with an Fe Dimer in a Magnetic Singlet State

**DOI:** 10.1021/acs.inorgchem.3c01775

**Published:** 2023-07-24

**Authors:** Emil H. Frøen, Peter Adler, Martin Valldor

**Affiliations:** † Centre for Materials Science and Nanotechnology (SMN), Department of Chemistry, University of Oslo, Sem Sælands vei 26, NO-0371 Oslo, Norway; ‡ Max-Planck-Institute for Chemical Physics of Solids, Nöthnitzer Straße 40, DE-01187 Dresden, Germany

## Abstract

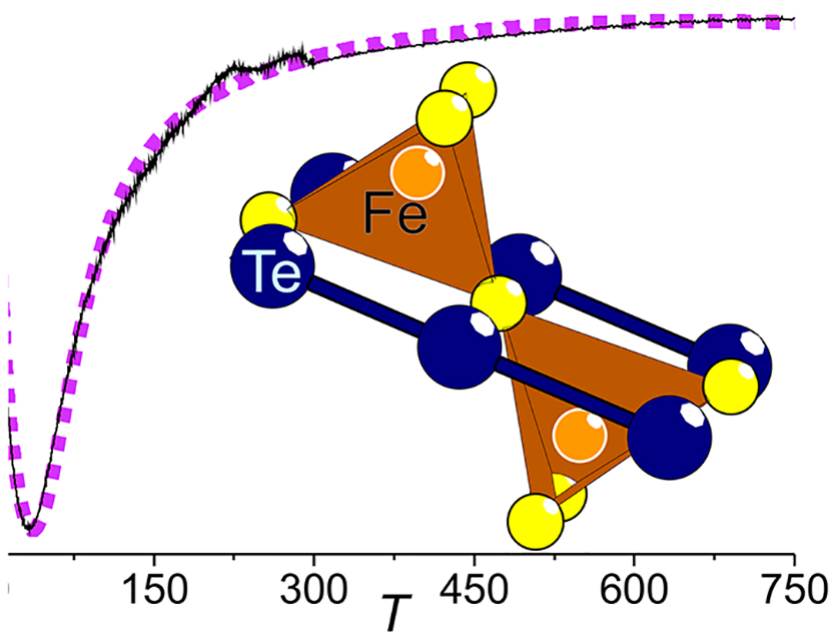

A new quaternary sulfide telluride, Ba_6_Fe_2_Te_3_S_7_, was synthesized by a solid-state reaction, and its crystal structure is novel. X-ray diffraction data on powder and single crystals reveal an orthorhombic lattice with *a* = 9.7543(3) Å, *b* = 18.2766(6) Å, and *c* = 12.0549(4) Å, and the noncentrosymmetric space group *Cmc*2_1_ (No. 36). The properties of the compound were studied by magnetic susceptibility investigations, specific heat measurements, Mössbauer spectroscopy, and density functional theory calculations. Assuming Ba^2+^ and, as verified by the Mössbauer spectra, Fe^3+^, the charge balance requires the presence of a polytelluride, suggested to be a straight-chain [Te_3_^4–^] polyanion. Further, the crystal structure contains [Fe_2_S_7_]^8–^ dimers of two vertex-sharing tetrahedra, with a nearly linear Fe–S–Fe atom arrangement. The dimer exhibits antiferromagnetic coupling, with a coupling constant *J* = −10.5 meV (*H* = −2*JS*_1_*S*_2_) and *S* = 5/2, resulting in a spin singlet ground state. The interdimer magnetic interaction is so weak that the magnetic dimers can be treated as individuals.

## Abstract 2

Polycrystalline Ba_6_Fe_2_Te_3_S_7_ can be synthesized by heating BaS with the corresponding amounts of elements in a closed ampule. In this purely inorganic crystal structure, two vertex-sharing Fe−S tetrahedrons constitute a magnetic Fe dimer that acts as a binuclear entity and enters a magnetic singlet ground state.

## Introduction

Iron is the fourth most abundant element in the Earth’s crust,^1^ and it is environmentally friendly and cheap. As such, the discovery that the element could form high-temperature superconductors was a matter of significant interest to the scientific community. Iron can, in fact, form superconducting compounds with heavier chalcogenides. FeSe is probably the most widely known example,^2^ but FeTe has also been shown to assume a superconducting state under strained conditions.^2,3^ Sulfur-based compounds are not known to superconduct under ambient conditions, but BaFe_2_S_3_ has been reported to undergo a superconducting transition under high pressure.^4^ Iron is the essential component in other superconductors as well, such as LiFeAs^5^ and NaFeAs,^6^ underlining its importance to fundamental science. Iron telluride also exhibits spin density waves along with several other spin arrangements, depending on the exact composition.^7,8^

As such, there is an incentive to search for novel compounds in iron chalcogenide systems. A relatively new approach, to this end, is the search for ordered multianionic compounds.^9,10^ According to Hume-Rothery, two distinct anionic species cannot form solid solutions if the ionic radii differ by more than approximately 15%. Beyond this limit, the two ions will assume distinct atomic sites.^11^ As the radius of Te is more than 15% larger than that of sulfur, telluride sulfides should contain ordering among the chalcogenide ions. Tellurides are also prone to forming polyanions of greatly varying lengths, ranging from simple pertelluride ions in FeTe_2_ to 15-chain polytellurides or infinite arrangements, making them a structural component with great potential for forming novel crystal structures.^12−14^ This makes iron bichalcogenide systems very interesting to investigate.

Here, we introduce a novel quaternary telluride sulfide, Ba_6_Fe_2_Te_3_S_7_, and report its synthesis, crystal structure, charge configuration, heat capacity, magnetic properties, and calculated properties, as obtained by density functional theory (DFT).

## Experimental Section

### Sample Preparation

All of the handling of the sample during the synthesis took place inside an argon-filled glovebox (H_2_O and O_2_ < 1 ppm). A stoichiometric mixture of about 0.5 g of BaS (Alfa Aesar, 99.7%), Fe (Alfa Aesar, 99%), and Te (Thermo Scientific, 99.99%) powders was homogeneously mixed in an agate mortar and pressed into a 13 mm diameter pellet with 3 tons of pressure. The resulting pellets were broken, and the fragments were placed in a corundum crucible. The crucible was sealed inside an evacuated silica ampule using an oxygen–hydrogen torch. Subsequently, the sample was placed in a muffle oven and heated to 700 °C at a 5 °C min^–1^ heating rate. The synthesis progressed at this temperature for 48 h before the furnace was turned off. The cooled sample was put back into the glovebox. All steps, including grinding, pelletization, sealing in a silica tube, and heating at 700 °C with subsequent cooling, were performed four times for a total of 192 h at 700 °C before the final sample was obtained. The single crystals, used for structure determination, were obtained from a similar synthesis with 46 h of heating but with a different target stoichiometry, Ba_9_Fe_4_Te_4_S_12_, while attempting a different synthesis.

The powder sample and single crystals of the title compound were specular black and appeared to be stable under ambient conditions. A minor Te (0.16 vol % from Rietveld refinement) impurity was observed in the powder X-ray diffraction (pXRD) pattern, but no other secondary phase was observed.

### X-ray Structure Determination

The pXRD data were obtained using a Bruker D8 Discover instrument with a Bragg–Brentano geometry and a Ge(111) Johanssen monochromator, Cu*K*α_1_ X-rays, and a Lynxeye detector. A zero-background-oriented silicon crystal XRD plate, covered with silicone grease, was used as the sample holder. An energy filter in the detector suppressed the fluorescence from Fe. The single-crystal data were collected at room temperature using a Bruker D8 Venture single-crystal diffractometer equipped with a Mo*K*α InCoatec microfocus X-ray source and a Photon 100 detector. The atomic structure was determined and refined using the JANA2006 software.^15^

### Physical Property Measurement System (PPMS)

The electric, magnetic, and heat capacity properties were determined using a Quantum Design PPMS. For the heat capacity measurements between 2 and 300 K, sintered polycrystalline pellets were used in the non-adiabatic thermal relaxation approach, and the sample was equilibrated at each temperature (15 min of extra waiting) before starting measurements. Apiezon N grease was used to attach the sample to the holder. Two sequential measurements were carried out at each temperature point, and the sample coupling never fell below 97% throughout the investigation. The magnetic measurements were performed on a powdered sample from a ground pellet in a polypropylene sample holder. To subtract a proper background signal, the empty holder was measured with the same parameters as those used for the sample measurement. For DC magnetization measurements, field-cooled (FC) protocols were carried out between 2 and 300 K with applied magnetic fields of 1, 3, and 6 T. A magnetization measurement was carried out at 300 K to determine the effect of ferromagnetic impurities, with applied fields from −7 to 7 T (data not shown). The electric conductivity of the sample was measured at room temperature, but the compound was found to be completely insulating within instrument measurement capacity (>2 MΩ).

For magnetic measurements above 300 K, a Lake Shore Cryotronics 7400-S Series VSM was utilized with an applied magnetic field of 1 T. The sample holder was made of BN, and the sample was held under a protective argon atmosphere during measurement.

### Scanning Electron Microscope (SEM) Imaging and Energy Dispersive X-ray (EDX) Analysis

SEM imaging and EDX analysis were carried out using a Hitachi SU 8230 Field Emission Gun Scanning Electron Microscope with an XFlash 6|10 EDX detector. A 15 keV acceleration voltage was used for both SEM and EDX. EDX analyses were carried out by determining the elemental composition of 13 separate crystallites and averaging the obtained values. The heaviest element, barium, was used as a reference point for the composition.

### Mössbauer Spectroscopy

^57^Fe Mössbauer spectra were collected at temperatures between 5 K and room temperature (295 K) using a standard WissEl spectrometer operated in the constant acceleration mode (^57^Co/Rh source) and a Janis SHI 850–5 closed-cycle refrigerator. About 30 mg of Ba_6_Fe_2_Te_3_S_7_ powder was mixed with BN and distributed in an acrylic glass sample container with an inner diameter of 13 mm. All isomer shifts are given relative to α-iron. The data were evaluated with the MossWinn program^16^ using the thin absorber approximation.

### Computational Details

The theoretical calculations were carried out using the Vienna Ab initio Simulation Package (VASP)^17,18^ utilizing the generalized gradient approximation (GGA) approach for the exchange-correlation energy, as formulated by Perdew-Burk-Ernzerhof (PBE).^19^ The calculations employed projected augmented wave (PAW) pseudopotentials^20^ with a plane wave energy cutoff of 400 eV and self-consistent field energy convergence criteria of 10^–6^ eV. To describe the strong correlation of the Fe-3d orbital electrons, a Hubbard *U*_eff_ repulsion term is added under the rotationally invariant Dudarev approach.^21^ A range of *U*_eff_ values from 0 to 6 eV were employed to observe how the parameter affected the description of the compound. Integrations over the Brillouin zone were carried out with a 4 × 2 × 3 gamma-centered sampling grid using the tetrahedron method with Blöchl corrections. Density of states (DOS) calculations were carried out with a doubled grid for integration of the Brillouin zone (8 × 4 × 6). The band structure path was determined with the Materials Cloud SeeK-path tool.^22,23^

An initial, noncollinear calculation incorporating spin–orbit coupling was carried out on a static structure relaxed nonmagnetically at *U*_eff_ = 0 eV to determine the alignment of the iron spin states relative to the structure and to investigate whether the ground state involves a noncollinear spin arrangement. These calculations utilized the same sampling grid used for the Brillouin zone. The use of symmetry was disabled for these calculations. As the ground state was found to be spin-collinear, all other calculations utilized a spin-collinear configuration. With one exception, the structures of all magnetic alignments were allowed to fully relax with respect to both unit cell and ionic positions until all forces were less than 10^–2^ eV Å^–1^. The calculation of magnetic couplings utilized static unit cells, which were structurally relaxed with the ground-state magnetic alignment.

Using the PBE0^24^ and HSE06^25^ functionals, hybrid functional calculations were carried out to determine the magnetic couplings, using only the gamma point for sampling the Brillouin zone and Gaussian smearing with a width of 0.02 eV. Otherwise, the parameters were the same as for the GGA calculations.

## Results

### Crystal Structure

**Figure 1:**
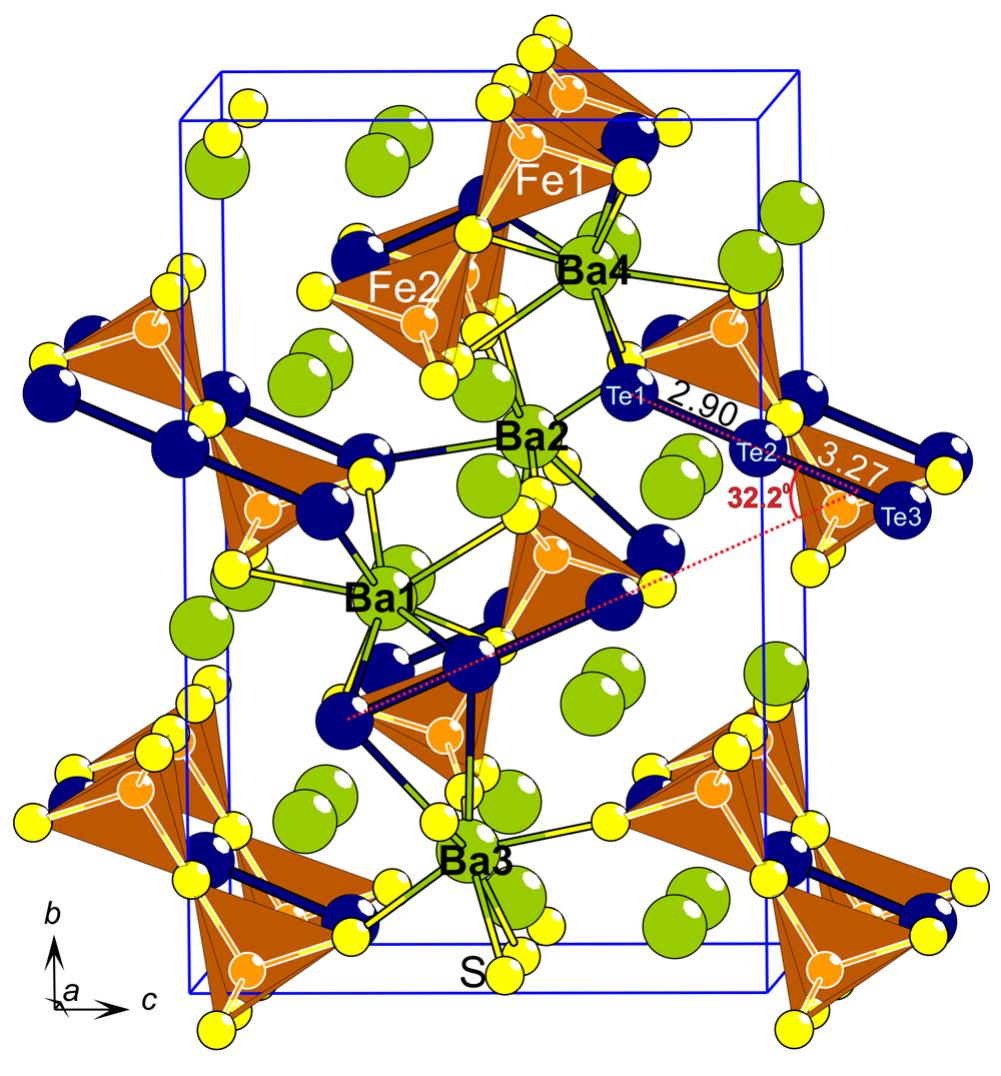
Extended unit cell content of the Ba_6_Fe_2_Te_3_S_7_ crystal structure. The dashed red lines represent the vector along which the telluride triplets are arranged, and the angle between these vectors is given. The Te–Te distances are given in Å.

The analysis of the single-crystal XRD data of Ba_6_Fe_2_Te_3_S_7_ at room temperature reveals a unique crystal structure, described in the *Cmc*2_1_ (No. 36) space group symmetry, with lattice constants *a* = 9.7522(5) Å, *b* = 18.093(1) Å, *c* = 12.0036(7) Å, *V* = 2117.9(2) Å^3^, and *Z* = 4 (Figure 1). The complete unit cell is given in Figure 1, and the refinement and structural parameters are given in Table 1 and Table 2, respectively.

**Table 1:** Single-Crystal Data Refinement Parameters for Ba_6_Fe_2_Te_3_S_7_

formula	Ba_6_Fe_2_Te_3_S_7_
radiation	Mo *K*α (λ = 0.71073 Å)
instrument	Bruker D8 Venture
physical appearance	specular black
crystal system	orthorhombic
space group	*Cmc*2_1_ (No. 36)
formula weight	1542.95
temperature (K)	293
*a* (Å)	9.7522(5)
*b* (Å)	18.093(1)
*c* (Å)	12.0036(7)
*V* (Å^3^)	2117.9(2)
*Z*	4
ρ_calc_ (g cm^–3^)	4.8389
no. of independent reflections	1827
no. of variables	97
GOF (all) on *F*^2^	1.67
*R*1 (obs) (%)	3.04
*R*1 (all) (%)	3.50
*wR*2 (obs) (%)	6.95
*wR*2 (all) (%)	7.09
CSD No.	2264569

**Table 2:** Ionic Positions of Ba_6_Fe_2_Te_3_S_7_, as Determined by Single-Crystal X-ray Diffraction

atom	site	*x*	*y*	*z*	*U* (Å^2^)
Ba1	8*b*	0.2809(1)	0.4360(1)	0.3252(1)	0.0166(3)
Ba2	4*a*	0	0.4185(1)	0.0275(1)	0.0162(4)
Ba3	4*a*	0	0.1632(1)	0.4844(1)	0.0115(3)
Ba4	8*b*	0.2495(1)	0.1829(1)	0.1868(1)	0.0181(3)
Fe1	4*a*	1/2	0.4723(2)	0.5965(3)	0.021(1)
Fe2	4*a*	1/2	0.2701(2)	0.4116(2)	0.021(1)
S1	4*a*	1/2	0.3045(3)	0.2326(5)	0.015(2)
S2	8*b*	0.3042(4)	0.2153(2)	0.4588(3)	0.022(2)
S3	8*b*	0.3066(4)	0.5311(2)	0.5587(3)	0.018(2)
S4	4*a*	1/2	0.3725(5)	0.5031(7)	0.031(2)
S5	4*a*	1/2	0.4342(4)	0.7755(5)	0.021(2)
Te1	4*a*	0	0.3120(1)	0.2728(2)	0.0193(5)
Te2	4*a*	0	0.3763(2)	0.4941(2)	0.0193(4)
Te3	4*a*	1/2	0.0518(1)	0.2437(2)	0.0215(5)

**Figure 2:**
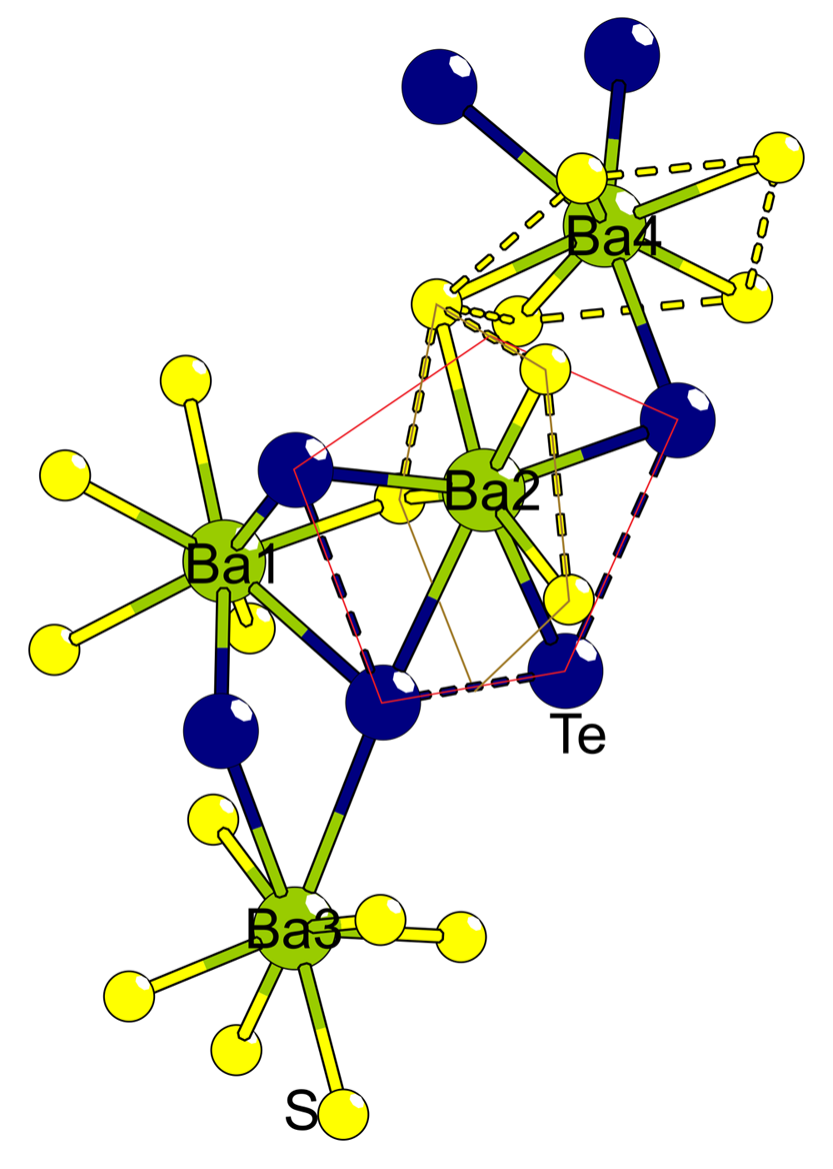
Barium ion coordinations. The Te–Te and S–S connections are to clarify coordination rather than bonding. The two polygons of thin lines represent the two mirror planes that penetrate the Ba2 site.

Each of the four distinct Ba^2+^ ions in the structure exhibits an individual coordination to sulfur and tellurium, with two, three, or four telluride neighbors (Figure 2). Ba2 and Ba3 assume a similar distorted-square, antiprismatic arrangement with four and two telluride coordinations, respectively. The even number of telluride ions allows the coordination to assume a symmetric arrangement of the anions. For Ba2, the position is bonded to four anionic species arranged separately on two perpendicular planes, similar to a *mer*-idional coordination, exhibiting an *mm*2 symmetry. The Ba3 arrangement is similar except that two tellurides are replaced by sulfur; the two remaining tellurides are situated adjacently. Ba1 is coordinated by three telluride ions and assumes a *fac* bicapped trigonal prismatic arrangement, with the telluride ions arranged on one side. Finally, Ba4 coordinates 3 Te and 5 S, but the spatial separation of Te by S can be represented as a 1+5+2 coordination, with the telluride ions occupying opposite sides of the barium ion and a five-coordinate belt of sulfur ions separating the unequally distributed telluride ions.

Three Te ions are arranged along an essentially straight vector parallel with the *bc*-plane, with a Te1–Te2–Te3 angle of 179.8(1)° (Figure 1). There are two different vectors the telluride chains follow within a single *bc*-plane, which differ by a 32.2° angle (Figure 1). The three tellurides have slightly different spacing among them, with Te1 and Te2 being 2.90 Å apart and Te2 and Te3 being 3.27 Å apart, indicating the presence of a pertelluride. The Te1–Te2 bond length is slightly longer than that of a pertelluride ion coordinated with barium (2.77 Å in BaTe_2_^26^) but about equal with the bonding in FeTe_2_ (2.90 Å^12^). Alternatively, the Te trimer row in the title compound may be a [Te_3_^4–^] polytelluride ion. In 2009, a review stated that no isolated, linear example of this species has ever been reported.^13^ The same review also remarked that species isoelectronic with [Te_3_^4–^], such as linear [I_3_^–^], may exhibit deviation from the ideal centrosymmetric distribution, which matches the observations for Ba_6_Fe_2_Te_3_S_7_. However, very recently, a centrosymmetric variation of the [Te_3_^4–^] ion was suggested in Ba_14_Si_4_Sb_8_Te_32_(Te_3_),^27^ having the same total length of the ion, which could indicate that it is a polytelluride ion. Alternatively, the [Te_2_^2–^][Te^2–^] straight chain is a coincident atomic arrangement. Beyond the trimer, the smallest Te–Te interatomic distance is at least 4.4 Å, so the chains would not behave as an extended polytelluride. The low electrical conductivity at room temperature corroborates this perspective.

**Figure 3:**
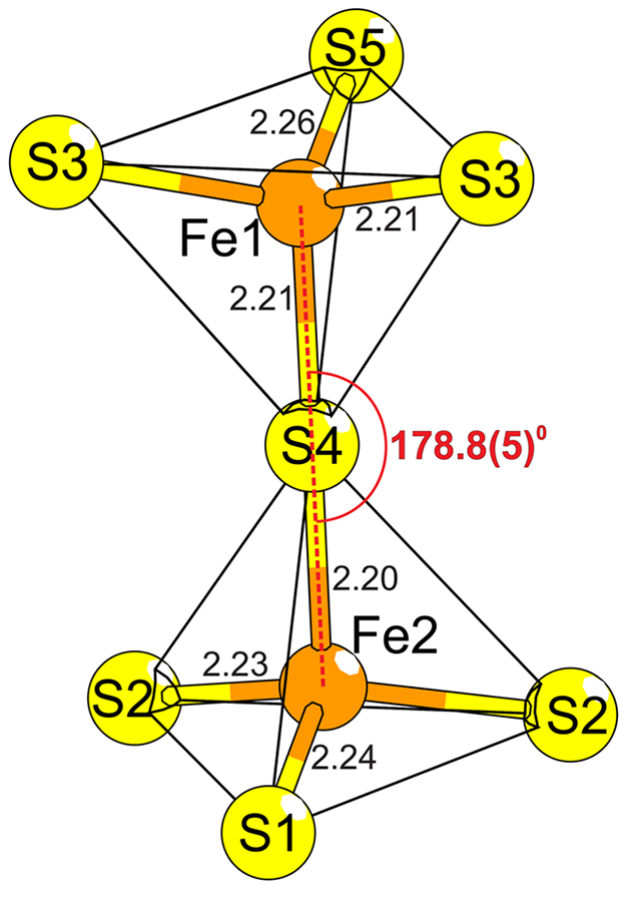
Dimeric structure of iron in Ba_6_Fe_2_Te_3_S_7_. Interatomic distances are given in Å.

The two distinct iron positions assume a vertex-sharing dimeric arrangement tetrahedrally coordinated with sulfur, forming an Fe–S–Fe angle of 178.8(5)°. The tetrahedra are arranged in a staggered configuration (Figure 3). It should be noted that other single-crystal measurements were refined to give an Fe–S–Fe angle even closer to 180°, including 179.6(6)° and 180.0(6)°. The central sulfide ion seems to be almost at an inversion symmetric point for the dimer, although this is not a symmetry in the suggested space group, and the Fe–S distances are slightly different between the two tetrahedra. The uneven spacing of the ions in the Te triplet also contributes to a different chemical environment between the two iron positions. The Fe–S–Fe dimers occupy the same *bc*-planes as the telluride chains, stacked alternatingly along the *a*-axis (Figure 1).

Edge-sharing Fe dimers, such as K_3_FeSe_3_,^28^ Rb_6_Fe_2_S_6_,^29^ and others,^29^ are known and characterized. For vertex-sharing arrangements, there are selected organic salts which exhibit transition metal elements in similar structures when bonded with transition metal (oxy-) halide anions such as [Fe_2_OBr_6_]^2–^, [Fe_2_OCl_6_]^2–^, and [Hg_2_I_7_]^2–^.^30−32^ The dimeric arrangements of Fe^3+^ consistently exhibit a singlet spin state.

### Powder XRD

**Figure 4:**
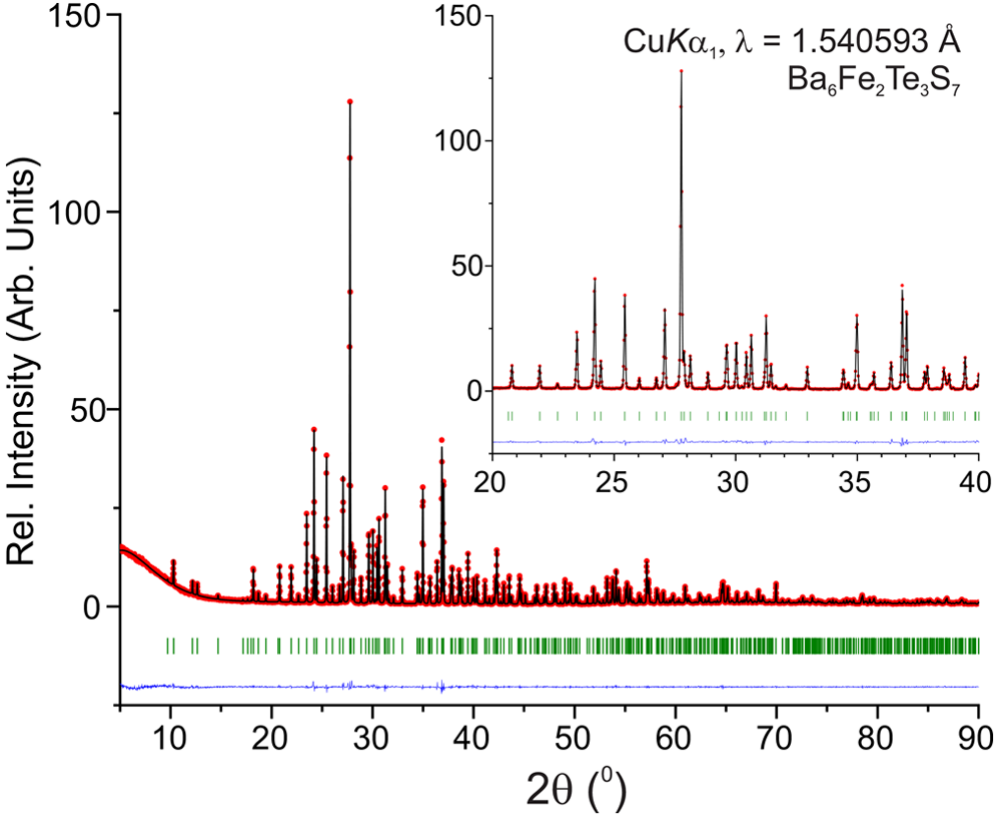
Rietveld refinement of Ba_6_Fe_2_Te_3_S_7_ pXRD data. The vertical lines (green) indicate the Bragg positions. Differences between the observed data (red) and the calculated data (black) are given in blue. The inset shows the fitting of the most prevalent peaks in more detail.

A refinement from pXRD yields more accurate lattice parameters compared to those from single-crystal measurements. Rietveld refinement (Figure 4) of the lattice parameters from the pXRD data yields *a* = 9.7543(3) Å, *b* = 18.2766(6) Å, and *c* = 12.0549(4) Å. Overall, the measured and calculated diffraction patterns match excellently.

### SEM and EDX Analysis

**Figure 5:**
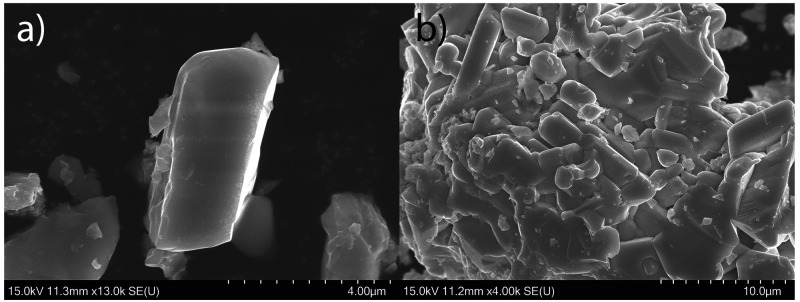
SEM images of (a) a single crystal of Ba_6_Fe_2_Te_3_S_7_ and (b) a larger cluster of crystallites. The scaling bars correspond to 4 and 10 μm, in (a) and (b), respectively.

Viewed in SEM, Ba_6_Fe_2_Te_3_S_7_ exhibits roughly rectangular prism crystal shapes, consistent with the expected mode of growth from the unit cell (Figure 5). EDX analysis gives the composition Ba_6.00(8)_Fe_2.06(9)_Te_3.06(4)_S_7.1(2)_, in excellent agreement with the determined crystal structure. Further, using the composition of Ba_6_Fe_2_Te_3_S_7_ in a synthesis resulted in an X-ray pure sample.

### Mössbauer Spectroscopy

**Figure 6:**
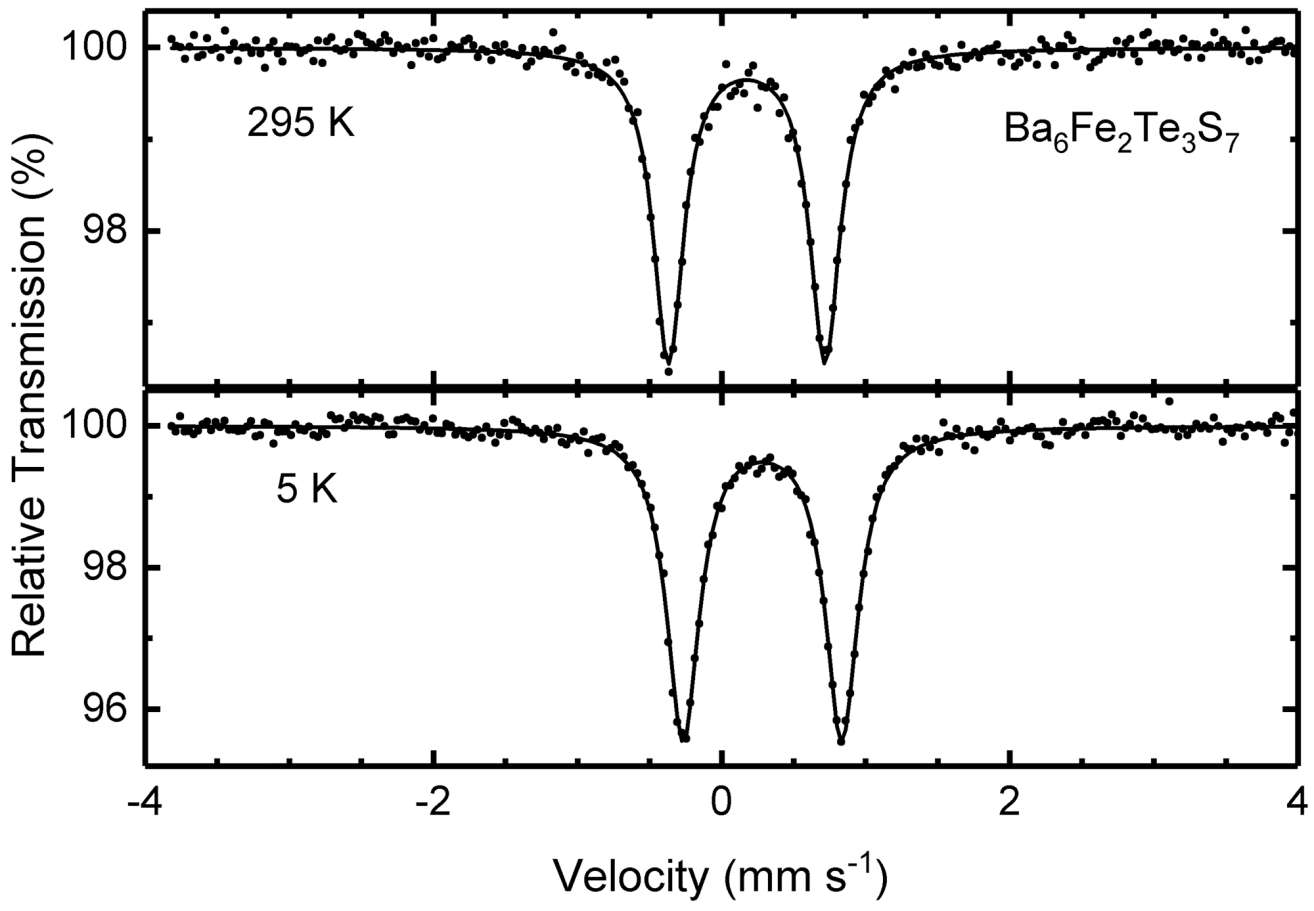
Mössbauer spectra of Ba_6_Fe_2_Te_3_S_7_ at room temperature and 5 K. Dots and solid lines correspond to the experimental and calculated spectra, respectively.

The room-temperature Mössbauer spectrum of Ba_6_Fe_2_Te_3_S_7_ consists of a single quadrupole doublet with an isomer shift (IS) = 0.174(1) mm s^–1^ and a quadrupole splitting (QS) = 1.086(3) mm s^–1^ (Figure 6). The crystal structure actually exhibits two distinct lattice sites for Fe; however, the difference in their local coordination geometries is too small to be resolved in the Mössbauer spectra. The quadrupole doublet persists despite cooling, and down to 5 K there are no signs of magnetic ordering or freezing. The Mössbauer parameters at 5 K are IS = 0.283(1) mm s^–1^ and QS = 1.101(2) mm s^–1^. The small isomer shift values are typical for Fe^3+^ in a tetrahedral environment;^33^ for example, IS = 0.16 mm s^–1^ at 300 K for KFeS_2_, and IS = 0.155 mm s^–1^ for Rb_6_[Fe_2_O_6_], an oxoferrate(III) with edge-sharing Fe dimers.^29^ Remarkably, the QS is quite large for Fe^3+^ where, for the half-filled *e*^2^*t*_2_^3^ configuration, only a lattice but no valence contribution to the electric field gradient occurs. As expected for Fe^3+^ and in contrast to Fe^2+^, the QS is nearly independent of temperature. The large QS can be attributed to the asymmetric local environment of Fe^3+^, which is due to the formation of dimers in the crystal structure.^29^ Thus, the compound can be formulated as either Ba_6_^2+^Fe_2_^3+^Te_3_^4–^S_7_^2–^ or Ba_6_^2+^Fe_2_^3+^Te_2_^2–^Te^2–^S_7_^2–^, in agreement with the crystal structure analysis indicating the presence of per- or polytellurides. Finally, it is noted that within the error limits of about 2%, there are no signs of Fe-containing impurities.

### Magnetic Properties

**Figure 7:**
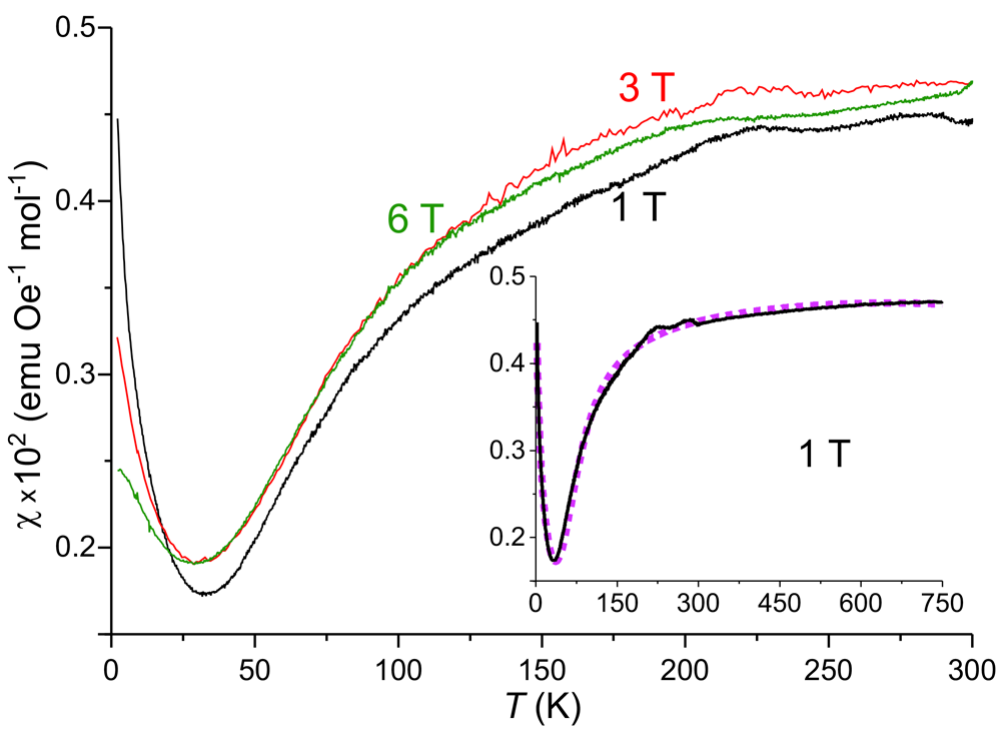
Field-cooled (FC) DC magnetic susceptibility of Ba_6_Fe_2_Te_3_S_7_ with temperatures in the range 2–300 K, for applied fields of 1, 3, and 6 T. The values shown in the figure are corrected for the contribution of ferromagnetic impurities, instrumental background from the sample holder, and the standard diamagnetic contribution of the atomic species present.^39^ The inset shows an extended curve, synthesized from two different measurements, ranging from 2–300 and 300–750 K, respectively, with an applied field of 1 T. The absolute value of the higher-temperature measurement has been shifted by simple addition to match the transition temperature with the lower-temperature measurement. The purple dashed curve in the inset is the Van Vleck fitting.

The magnetic susceptibility of Ba_6_Fe_2_Te_3_S_7_ corresponds to neither a typical (anti)ferromagnetic behavior nor a paramagnetic behavior (Figure 7). The majority of the 2–750 K temperature range is dominated by an apparent asymptotic increase in susceptibility with increasing temperature, although it should be noted that the reliability of the absolute values for the susceptibility measured in the 300–750 K region is lower than that of the measurements in the low-temperature region. The high-temperature values in the Figure 7 inset have been shifted by simple addition to ensure continuous overlap with the low-temperature measurements. The increase in susceptibility at the lowest temperatures is probably due to paramagnetic iron impurities.

There are a number of irregularities in the curves, but the origin of these cannot be specified. Due to the small absolute scale of the susceptibility, even marginal effects from impurities would appear as major contributions, making the assignment of any meaning to these anomalies uncertain. At least some of the irregularities, particularly in the 200–300 K range, are due to instrumental errors.

Considering the structural motif of iron dimers and the results from Mössbauer spectroscopy, it can be assumed that the shape of the magnetic susceptibility curve of Ba_6_Fe_2_Te_3_S_7_ reflects antiferromagnetic coupling of the *S* = 5/2 (Fe^3+^) centers within the dimers via the bridging S^2–^ ions, which leads to a diamagnetic singlet ground state (*S*′ = 0).

The magnetic properties of the dimeric magnetic species are typically described by the isotropic spin-exchange Hamiltonian *H* = −2*JS*_1_·*S*_2_. Using the Van Vleck formula (eq 1) with *S*_1_ = *S*_2_ = 5/2 affords^28,32,34−37^
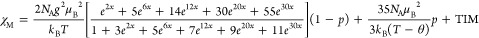
1where , *N*_A_ is the Avogadro constant, *g* is a *g*-factor (fixed to 2 in our fitting to avoid overparameterization), μ_B_ is the Bohr magneton, *k*_B_ is the Boltzmann factor, *T* is the temperature in Kelvin, *p* is the fraction of magnetic Fe^3+^ impurities, and *J* is the magnetic coupling constant. TIM is a temperature independent of paramagnetic contribution. Exponential parts of the equation correspond to the thermal population of the excited spin states of the dimer with *S*′ = 1, 2, 3, 4, and 5, respectively.

The Van Vleck equation provides an adequate fit to the measured data (inset of Figure 7) over the entire temperature range, although there is a consistent mismatch for the location of the susceptibility minimum between the fit and the measurement, with the fitted minimum always located at a slightly higher temperature. The obtained value for the coupling constant *J* is −10.5(2) meV or −85(2) cm^–1^, averaged between three measurements at 1, 3, and 6 T. This value is comparable to the coupling constants of −14.2 and −10.4 meV reported for the edge-sharing dimers in the compounds Na_3_FeS_3_ and Cs_3_FeS_3_, respectively.^37^ The overall shape and magnitude of the curves reported in the same paper correspond well with those observed here. The fitted θ and *p* values are field-dependent due to saturation of the paramagnetic contribution. The obtained *p* values range from about 0.8% to 1.9%, increasing with the applied magnetic field.

### Heat Capacity

**Figure 8:**
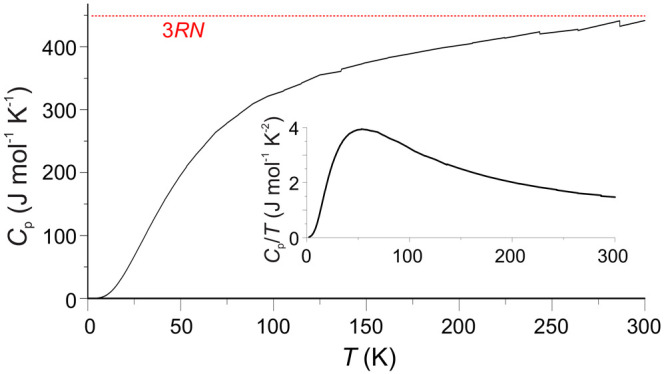
Specific heat measurements of Ba_6_Fe_2_Te_3_S_7_, with the Dulong-Petit limit inserted in red. The inset depicts the specific heat divided by the temperature vs temperature.

The heat capacity of Ba_6_Fe_2_Te_3_S_7_ exhibits no distinct features beyond discontinuities that we assign to minor measurement errors (Figure 8). There is no indication that the compound undergoes a common long-range magnetic or structural transition.

### Density Functional Theory (DFT)

The initial consideration that the magnetic structure might be noncollinear at 0 K was extracted from the alignment of the iron positions. Comparison of collinear and noncollinear arrangements with spin–orbit coupling effects indicated that the collinear, antiferromagnetic arrangement of spins is the more stable configuration. The difference in energy between the collinear and noncollinear arrangements was 3.4 meV per dimer.

**Figure 9:**
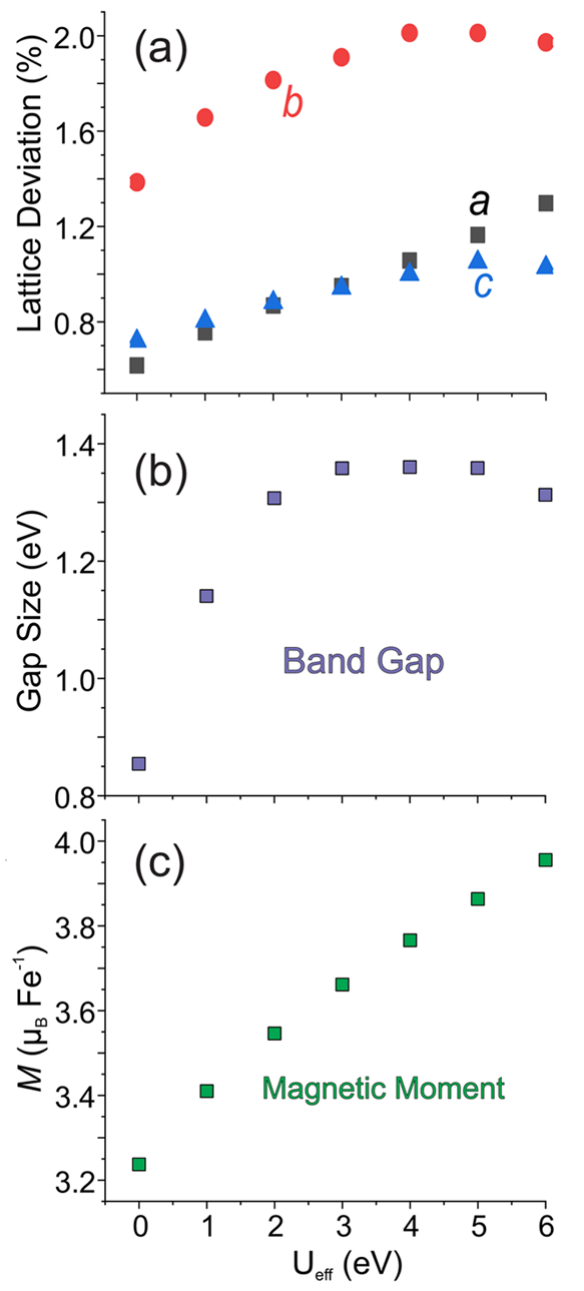
(a) The difference between the calculated and experimental lattice parameters with a varying Hubbards parameter, expressed in terms of percentage deviation. (b) The band gap of Ba_6_Fe_2_Te_3_S_7_ with variation in *U*_eff_. (c) The local magnetic moment on the Fe ion with variation in *U*_eff_.

The overall agreement of the relaxed unit cells with the pXRD refinement lattice parameters is exceptional (Figure 9a). With *U*_eff_ = 0 eV, only the *b*-parameter differs from the experimental values by more than 1%, although the calculated values consistently overestimate the lattice parameters. Applying the +U correction caused the *a*-parameter to increase nearly linearly across the full *U*_eff_ range, while the *b*- and *c*-parameters plateau at *U*_eff_ values of 4–5 eV.

Bader charge analysis of the telluride ions shows that the number of electrons associated with each position in the triplet chains is not an even distribution or a distinct pertelluride–telluride pairing. Rather, the terminal telluride positions are associated with similar electron charges, while the central position is associated with significantly fewer electron charges. Utilizing the Bader charge distribution of BaTe and BaTe_2_ as references for tellurides and pertellurides, respectively, one finds that the central telluride position is associated with approximately the equivalent charge of a pertelluride species. The terminal telluride positions are associated with an intermediate amount of charge between telluride and pertelluride characters. With a linear interpolation between the pure telluride and pertelluride extremes, the terminal telluride positions exhibit roughly equal character of both. Interpreting the character as valency and summing between the tellurides in each triplet, this equals the associated charge that one would expect from a total valency of −4, which is the value one should expect. The comparatively similar charges of the terminal positions strongly suggest a three-chain polytelluride. If the chains were formed from a discrete pertelluride–telluride pair, [Te_2_^2–^][Te^2–^], the terminal positions should exhibit a substantial difference in character. The difference in the calculated valence is roughly 11%, with the more isolated ion being associated with a greater charge. This behavior is largely independent of the *U*_eff_ parameter and occurs with both hybrid functionals. As such, the Bader analysis may be considered to provide substantial support toward the view of the [Te_3_^4–^] polytelluride description.

Ba_6_Fe_2_Te_3_S_7_ is found to be a semiconductor, although the nature of the band gap varies with the *U*_eff_ parameter. As the *U*_eff_ value exceeds 2 eV, the band gap changes character and becomes comparatively independent of *U*_eff_ (Figure 9b). The obtained band gaps range from 0.85 to 1.36 eV for the GGA+U approach.

The local magnetic moment of the Fe ions ranges from 3.2 to 4.0 μ_B_, increasing nearly linearly with the *U*_eff_ parameter (Figure 9c). This high value agrees with the high-spin state of Fe^3+^. The values displayed in the figure are averages, as the two iron ions in the dimer are not exactly equal due to the lack of symmetry to enforce an identical environment. The calculated magnetic moments for the Fe ions in one dimer differ by 0.030 to 0.017 μ_B_ as *U*_eff_ varies from 0 to 6 eV, respectively.

**Figure 10:**
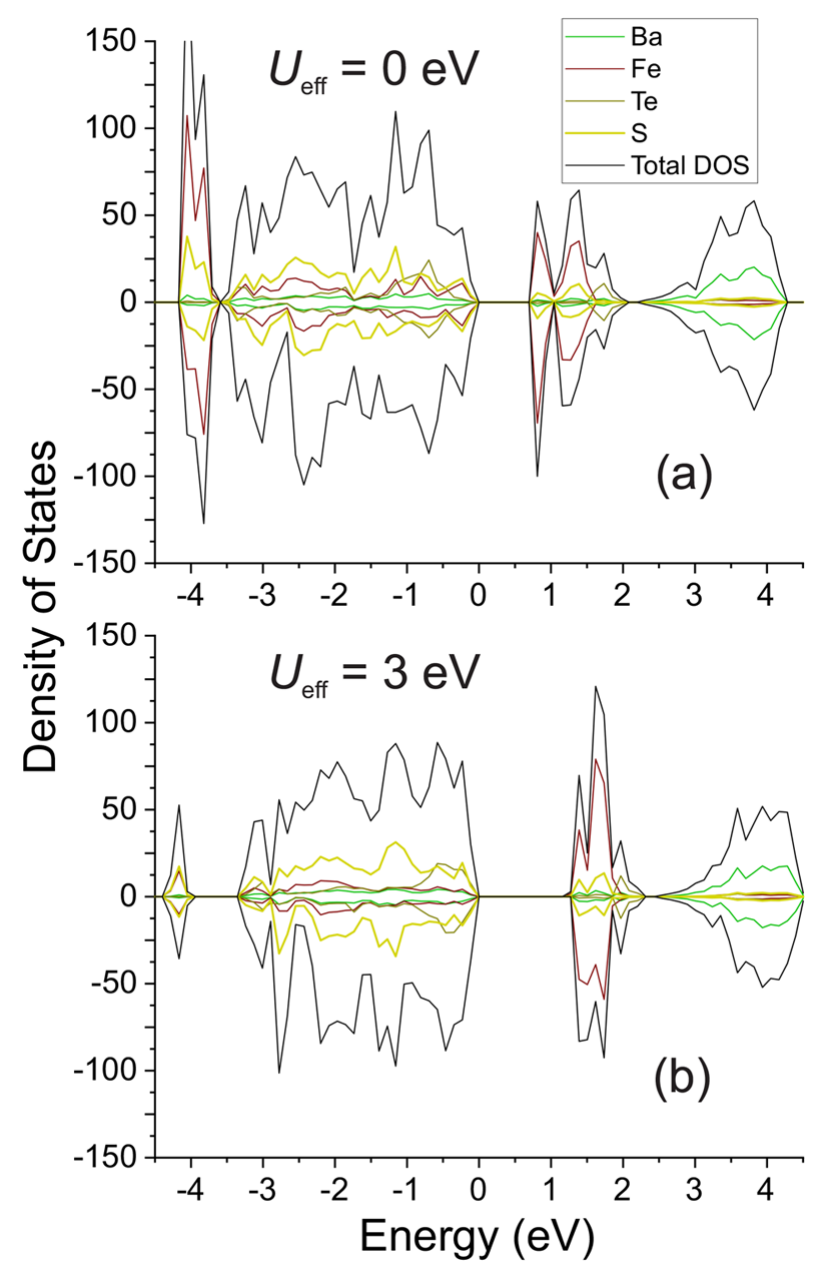
DOS of Ba_6_Fe_2_Te_3_S_7_, calculated with (a) *U*_eff_ = 0 eV and (b) *U*_eff_ = 3 eV.

The change in the bandwidth of *U*_eff_ is accompanied by a change in the nature of the gap transition. When *U*_eff_ is between 0 and 2 eV, the transition is of mixed character between a Mott insulator and a charge transfer insulator. The valence band (VB) maximum is composed of Fe-3d orbitals along with S-3p orbitals with roughly equal contributions, while the conduction band (CB) minimum is predominantly of Fe-3d character (Figure 10a).

At *U*_eff_ values exceeding 2 eV, the composition of the edges of the band gap changes and the influence on the valence-band-maximum (VBM) by the Fe-3d orbitals is greatly diminished. Instead, the transition approaches a pure charge transfer-like character, with equal contributions from S-3p and Te-5p orbitals. Further increase in the *U*_eff_ parameter shifts the VBM character further toward a Te-5p composition (Figure 10b).

**Figure 11:**
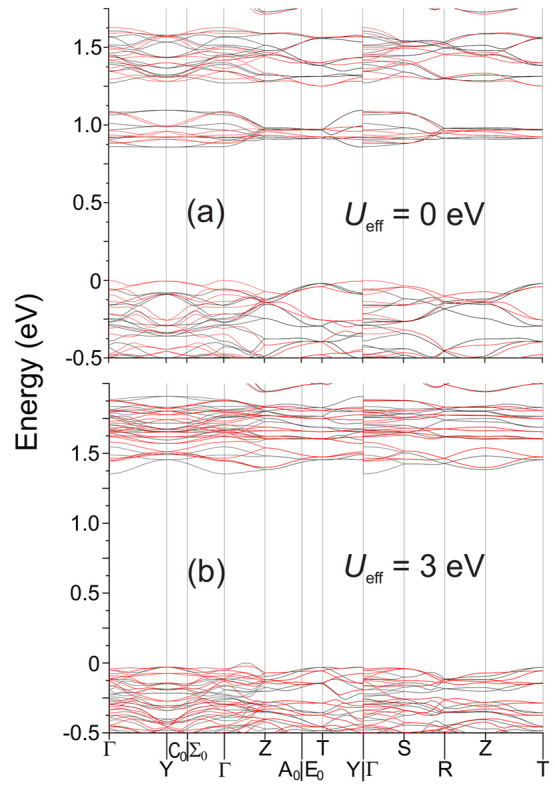
Band structure of Ba_6_Fe_2_Te_3_S_7_, calculated with (a) *U*_eff_ = 0 eV or (b) *U*_eff_ = 3 eV. The black bands represent spin-up states, and the red bands represent spin-down states. The energy scale is relative to the Fermi energy.

A feature of the band structure that occurs for all tested values of *U*_eff_ is that the electronic states are spin-dependent (Figure 11). For *U*_eff_ = 0 eV, this results in the lowest energy transitions involving spin reversal. The lowest energy transitions are the Γ–Y and Γ–Γ transitions, which have nearly equal energy. For transitions without spin reversal, the indirect T–Y transition is the smallest, with a gap of 0.88 eV.

At *U*_eff_ = 3 eV, the lowest energy transition occurs from an off-symmetry position in the VBs, between Γ and Z, to an Γ position in the CB. Notably, the entirety of the lowest energy CB states, as well as the VB maximum, are composed of electronic states with a single spin.

The most favorable magnetic interaction remains constant throughout the range of *U*_eff_ values. Singlet-arranged dimers are where the paired Fe ions exhibit spins in opposite directions. Energy contributions from interdimeric magnetic interactions are marginal and can be disregarded. A diamagnetic, low-spin configuration is enormously unfavorable, with energy levels that are about 2–4 eV per dimer higher compared to the high-spin configuration (depending on the *U*_eff_ employed), further suggesting that the system is not in a low-spin Fe^2+^ configuration. The most favorable long-range collinear antiferromagnetic configuration with iron in alternating *bc*-planes exhibits opposite spins, and the ferromagnetic spin arrangements are 0.48 and 0.54 eV per dimer, respectively, above the lowest energy state, with *U*_eff_ = 0 eV. These values are notably quite similar, suggesting that the interdimeric coupling is much weaker than the intradimer coupling.

The typical approach for determining the coupling constant *J* of dimeric species in DFT is the broken symmetry approach. Based on the same spin-exchange Hamiltonian *H* = −2*JS*_1_·*S*_2_, as defined in the experimental description, the coupling constant, per dimeric pair, may be determined from the difference in energy between singlet and triplet states^40^
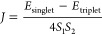
2

The coupling constant obtained from this equation is dependent on the accuracy of the exchange-correlation functional employed. For *S*, either the ideal value or the DFT calculated value may be employed. For high-spin Fe^3+^ with *S* = 5/2, eq 2 becomes
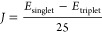
3

Using the ideal spin value (5/2) for Fe^3+^, the coupling constant comes out in the range of −24.9 to −8.9 meV, decreasing with a higher +*U*_eff_ parameter. The hybrid calculations give coupling constants of −14.3 and −14.4 meV for PBE0 and HSE06, respectively. The closest approximation to the hybrid calculations is obtained with +*U*_eff_ = 3 eV, giving a coupling constant of 14.2 meV. The hybrid results exhibit decent agreement with the experimental fittings but overestimate the magnitude by about 36–37%. DFT is known to substantially overestimate the magnitude of magnetic coupling energies, with the scope of error potentially up to two- to four-fold the experimental value,^41−43^ although literature on calculations with dimeric sulfur-based compounds is scarce. Studies suggest hybrid functionals provide significantly more accurate coupling constants, with HSE and PBEh providing coupling constants with a mean absolute percentage error of about 10% for organic compounds with Fe dimers.^40^

In general, the model corroborates the experimental expectation of a singlet state. Analysis of the spin density of the singlet system shows that the system exhibits superexchange mediated via a p orbital on the shared-vertex sulfide ion.

## Discussion

The different analyses of Ba_6_Fe_2_Te_3_S_7_ give a coherent picture of the origin of its unusual susceptibility data. Mössbauer spectroscopy data and DFT calculations both agree that Fe in Ba_6_Fe_2_Te_3_S_7_ is in a trivalent high-spin state, with antiferromagnetic coupling rendering the total spin state of each dimer a singlet at low temperatures. In effect, the bulk material exhibits a very low magnetic susceptibility, despite exhibiting strong electronic correlations. The lack of magnetic coupling between the spin dimers, as suggested by DFT calculations, agrees with the Mössbauer spectroscopy and heat capacity, indicating the absence of magnetic long-range ordering down to 2 K. The temperature dependence of the magnetic susceptibility can be described reasonably well over the whole measured temperature range of 2–750 K, if isotropic exchange between the *S* = 5/2 Fe^3+^ centers within the dimer is assumed and the corresponding Van Vleck equation is used. The pronounced antiferromagnetic coupling with an exchange constant of −10.5 meV (85 cm^–1^) is a consequence of superexchange through the central sulfide ion, as per the Goodenough-Kanamori rule,^44,45^ and combined with the high magnetic isolation of the diamagnetic matrix, each dimer essentially behaves like a binuclear entity.

The exact nature of the telluride triplets in the structure is difficult to assign accurately from experimental data due to the *sliding-scale* nature of polytellurides. Among the charge balancing, the trivalent Fe ions, and the interatomic distances, the shorter telluride–telluride distance may be confidently established as a feature of a pertelluride. Whether the third telluride in the chain is part of a [Te_3_^4–^] unit is likely true because of the recently observed centrosymmetric [Te_3_^4–^] unit. Bader charge analysis provides a case toward the [Te_3_^4–^] description, with a similar charge assignment on the terminal positions. While the distortion from a centrosymmetric chain likely gives the tellurides a mixed nature between [Te_2_^2–^][Te^2–^] and [Te_3_^4–^], the latter is deemed to be a better description for the smaller difference in charge character between the terminal positions. Thus, the most reliable representation of the compound charge distribution is (Ba^2+^)_6_(Fe^3+^)_2_(Te_3_)^4–^(S^2–^)_7_.

## Conclusion

Powder and single crystals of Ba_6_Fe_2_Te_3_S_7_ were prepared by solid-state synthesis, heating a mixture of BaS, Fe, S, and Te. Characterization of the compound by structural determination, magnetic and heat capacity measurements, Mössbauer spectroscopy, and DFT analysis suggests the presence of trivalent iron arranged in dimers with a spin-singlet ground state. The telluride component of the compound was determined to constitute a straight-chain [Te_3_^4–^] polyanion. The compound was measured to be an electric insulator at room temperature, which was confirmed by DFT analysis of the band structure.
